# Supplementary material for: A Comprehensive Analysis of Neoadjuvant Chemotherapy in Breast Cancer: Adverse Events, Clinical Response Rates, and Surgical and Pathological Outcomes—Bozyaka Experience
Source: Cancers (Basel). 2025 Jan 7;17(2):163. doi: 10.3390/cancers17020163 (PMC11763700; doi:10.3390/cancers17020163)
Supplement: Supplementary file 1 [file cancers-17-00163-s001.zip › cancers-3414182-supplementary.pdf]

Table S1a. General pathologic response.

General Pathological Response (Breast and Axilla)	a) Overall patients cN_{ax}(0-3) (n=229)			b) Clinically Axillary LN(+) cN_{ax}(1-3) (n=213)			c) Axilla LN Bx; malignant pN_{ax}(+) (n = 129)		
	pCR	*non-pCR*	*p**	*pCR*	*non-pCR*	*p**	*pCR*	*non-pCR*	*p**
Molecular subtypes	*%(n)*	*%(n)*		*%(n)*	*%(n)*		*%(n)*	*%(n)*	
Luminal A	0% (0)	100% (23)	*.005* [cc]	0% (0)	100% (23)	*.006* [cc]	0% (0)	100% (15)	*.012* [f]
LB-HER2(-)	15% (15)	85% (88)	*<.001*	13% (13)	87% (84)	*<.001*	9% (5)	91% (51)	*<.001* [cc]
LB-HER2(+)	45% (21)	55% (26)	.003 [cc]	44% (18)	56% (23)	*.006* [cc]	39% (12)	61% (19)	*.092*
HER2 enriched	65% (13)	35% (7)	*<.001* [cc]	65% (13)	35% (7)	*<.001* [cc]	71% (10)	29% (4)	*<.001* [cc]
Triple-negative	33% (12)	67% (24)	.433 [cc]	34% (11)	66% (21)	*.327* [cc]	46% (6)	54% (7)	*.094* [f]
Total	27% (61)	73% (168)	*<.001*	26% (55)	74% (158)	*<.001*	26% (33)	74% (96)	*<.001*

For HER2(+) Diseases	*pCR*	*non-pCR*	*p**	*pCR*	*non-pCR*	*p**	*pCR*	*non-pCR*	*p**
Trastuzumab	30% (8)	70% (19)	.007 [cc]	30% (8)	70% (19)	*.005* [cc]	28% (5)	72% (13)	*.045* [cc]
Dual anti-HER2	67% (26)	33% (13)		70% (23)	30% (10)		63% (17)	37% (10)	
Total	51% (34)	49% (32)		52% (31)	48% (29)		49% (22)	51% (23)	

Note. *: Chi-Square Test, f: fisher's Exact Test, cc: continuity correction,
Abbreviations: Bx: Biopsy, pCR: pathological Complete Response, cT: clinical Tumor Stage, cNax: clinical Axillary Lymph Node Stage, pNax: Histopathologically, axillary lymph node was shown to be malignant

Table S1b. Breast tumor pathologic response.

Molecular Subtypes	pCR		non-pCR			non-pCR: MRD		non-pCR: Partial Response		non-pCR: Unresponsive	
	%	*n*	%	*n*	*p**	%	*n*	%	*n*	%	*n*
Luminal A, (n = 23)	0%	0	100%	23	*0.003* [cc]	*0%*	*0*	*61%*	*14*	*39%*	*9*
LB - HER2(-), (n = 103)	16%	16	84%	87	*<0.001*	*9%*	*9*	*57%*	*59*	*18%*	*19*
LB - HER2 (+), (n = 47)	51%	24	49%	23	*<0.001* [cc]	*17%*	*8*	*28%*	*13*	*4%*	*2*
HER2 enriched (n = 20)	65%	13	35%	7	*<0.001* [cc]	*15%*	*3*	*15%*	*3*	*5%*	*1*
Triple negative (n = 36)	33%	12	67%	24	*0.606* [cc]	*3%*	*1*	*39%*	*14*	*25%*	*9*
Total (n = 229)	28%	65	72%	164	*<0.001*	*9%*	*21*	*45%*	*103*	*18%*	*40*

Note. *: Chi-Square Test, cc: continuity correction.
Abbreviations: pCR: pathological Complete Response, MRD: Minimal Residual Disease

Table S1c. Axillary lymph node(s) pathologic response.

Molecular Subtypes	All patients (cN_{ax} 0-3); (n=229)					Clinical axillary LN(+) patients (cN_{ax} 1-3); (n= 213)					Axilla LN bx; malignant pN_{ax}(+); (n= 129)				
	pCR		non-pCR		*p**	pCR		non-pCR		*p**	pCR		non-pCR		*p**
	%	n	%	n		%	n	%	n		%	n	%	n	
Luminal A	0%	0	100%	23	*<0.001* [cc]	0%	0	100%	23	*<0.001* [cc]	0%	0	100%	15	*0.004* [cc]
LB-HER2 (-)	25%	26	75%	77	*<0.001*	27%	26	73%	71	*<0.001*	18%	10	82%	46	*<0.001* [cc]
LB-HER2 (+)	55%	26	45%	21	*0.012* [cc]	63%	26	37%	15	*0.003* [cc]	55%	17	45%	14	*0.034* [cc]
HER2 enriched	95%	19	5%	1	*<0.001* [cc]	95%	19	5%	1	*<0.001* [cc]	100%	14	0%	0	*<0.001* [cc]
Triple negative	47%	17	53%	19	*0.320* [cc]	53%	17	47%	15	*0.202* [cc]	54%	7	46%	6	*0.231* [f]
Total, % - n	38%	88	62%	141	*<0.001*	41%	88	59%	125	*<0.001*	37%	48	63%	81	*<0.001*

Note. *: Chi-Square Test, cc: continuity correction.
Abbreviations: cNax: clinical Axillary Lymph Node Stage, pCR: pathological Complete Response, pNax: (+): pathologically Malignant Axillary Lymph Node
